# Correlation versus hybridization gap in CaMn$_{2}$Bi$_{2}$

**DOI:** 10.1038/s41598-023-35812-2

**Published:** 2023-06-07

**Authors:** Christopher Lane, M. M. Piva, P. F. S. Rosa, Jian-Xin Zhu

**Affiliations:** 1 Theoretical Division, Los Alamos National Laboratory, Los Alamos, NM 87545 USA; 2 Center for Integrated Nanotechnologies, Los Alamos National Laboratory, Los Alamos, NM 87545 USA; 3 Max Planck Institute for Chemical Physics of Solids, Nöthnitzer Str. 40, 01187 Dresden, Germany; 4 Institudo de Física “Gleb Wataghin”, UNICAMP, Campinas, SP 13083-859 Brazil; 5 Division of Materials Physics and Application, Los Alamos National Laboratory, Los Alamos, NM 87545 USA

**Keywords:** Electronic properties and materials, Magnetic properties and materials, Structure of solids and liquids, Structural properties, Two-dimensional materials

## Abstract

We study the interplay between electronic correlations and hybridization in the low-energy electronic structure of CaMn$_2$Bi$_2$, a candidate hybridization-gap semiconductor. By employing a DFT+*U* approach we find both the antiferromagnetic Néel order and band gap in good agreement with the corresponding experimental values. Under hydrostatic pressure, we find a crossover from hybridization gap to charge-transfer insulting physics due to the delicate balance of hybridization and correlations. Increasing the pressure above $P_c=4$ GPa we find a simultaneous pressure-induced volume collapse, plane-to-chain, insulator to metal transition. Finally, we have also analyzed the topology in the antiferromagnetic CaMn$_2$Bi$_2$ for all pressures studied.

## Introduction

The electronic structure of fermionic correlated systems is driven by the competition between the tendencies of the electron to spread out as a wave and to localize as a particle, the latter usually accompanied with magnetism. That is, the interplay of the spin and charge degrees of freedom is a central issue^1^. Layered two-dimensional (2D) materials provide a unique platform for studying this dual nature of the electronic states which produces rich phase diagrams including high temperature superconductivity^2–4^, non-trivial topological insulating and semi-metallic phases^5^, quantum spin liquid states^6^, and strange metal behavior^7^.

In particular, the iron-based superconductors have been under vigorous experimental and theoretical study since the discovery of unconventional high-temperature superconductivity in La[O$_{1-x}$F$_x$]FeAs in 2008^8^. Since then a family of compounds with related layered crystal structures and chemical compositions were discovered including FeSe, LiFeAs, *R*FeAsO (*R* = rare earth), *A*Fe$_2$As$_2$ (*A* = Ca, Sr, Ba, Eu), termed the ‘11’, ‘111’, ‘1111’, and ‘122’ type structures, respectively^9^, The highest superconducting transition temperature of 56 K has been found in the 1111-type compound Gd$_{0.8}$Th$_{0.2}$FeAsO^10^.

To enhance the superconducting transition temperature and search for new broken symmetry phases, Fe was substituted away and replaced by other transition metals such as Cr, Mn, Co, and Ni. These isostructural compounds form new ground states including metallic (Co-based), itinerant antiferromagnetic (Cr-based), superconducting (Ni-based), and semiconducting antiferromagnetic (Mn-based) behavior. The Mn-based pnictides garnered special interested due to their similarity to the phenomenology of the high-temperature cuprate superconductors. In particular, the Mn-based compounds exhibit insulator-metal transitions upon either doping or application of pressure, but superconductivity has yet to be reported^11–17^, even though pressure induced superconductivity is observed in other Mn-based materials^18,19^. In general this suggests that the manganese pnictides possibly form a bridge between the pnictide and cuprate material families.

Recent experimental and theoretical studies find CaMn$_2$Bi$_2$ to host many intriguing properties including large anisotropic magnetoresistance^20^ and a plane-to-chain structural transition^21^. Most intriguingly, it has been suggested that CaMn$_2$Bi$_2$ may be a hybridization-gap semiconductor^22,23^. In line with this claim, low-temperature electrical transport measurements find a slight increase of the gap under pressure^24^. This type of behavior is akin to Ce$_3$Bi$_4$Pt$_3$ and other heavy fermion compounds^25–27^. Therefore, CaMn$_2$Bi$_2$ could provide a link between the cuprates, pnictides, and heavy fermion systems.

In this article, we present a first-principles investigation of the electronic and magnetic structure of CaMn$_2$Bi$_2$. We find the delicate balance between electronic correlations and hybridization to depend sensitively on pressure, resulting in a non-monotonic behavior of the band gap. In the pristine case, we are able to obtain an accurate ground state by including an effective Hubbard *U*, which significantly improves the agreement with experiments over earlier theoretical studies where GGA-PBE predicts a metal^22^, while hybrid functional dramatically overestimates the gap by an order-of-magnitude^24^. The good agreement also provides an important starting point for the study of pressure effects. Under applied hydrostatic pressure, we find CaMn$_2$Bi$_2$ to behave as a hybridization gap material up to 3 GPa and a correlation driven compound for larger pressures. Most strikingly, we find a large volume collapse due to a plane-to-chain structural transition at $P_c=4$ GPa, which simultaneously produces a metallic ground state. Moreover, the spin (orbital) manganese magnetic moments significantly decrease (increase) across the critical pressure. The predicted critical pressure and volume collapse are in good accord with experimental values^21^. Finally, we also find the antiferromagnetic CaMn$_2$Bi$_2$ to be topologically trivial for all studied pressures.

## Results

### Magnetic and electronic structure

**Figure 1:**
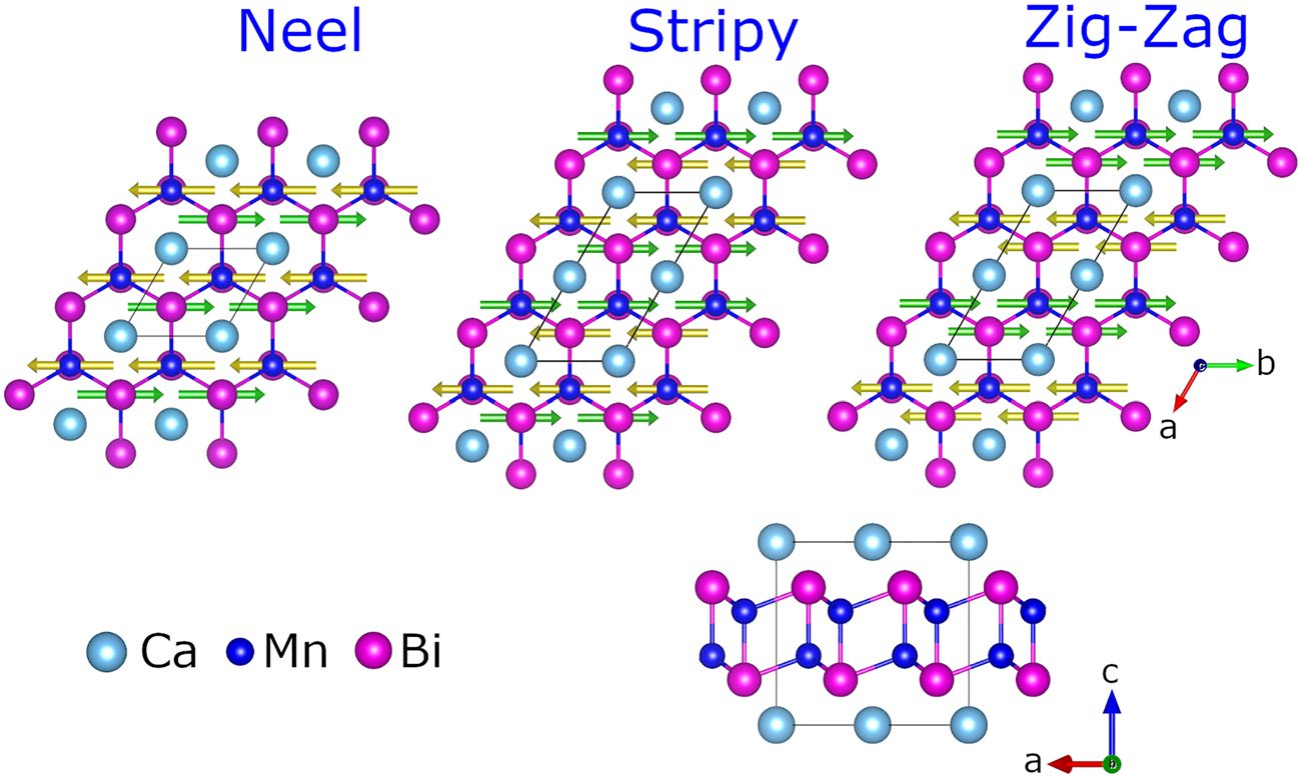
Various antiferromagnetic ground state configurations within the crystal structure of CaMn$_2$Bi$_2$. Green (gold) arrows represent the positive (negative) manganese magnetic moments. The stacking of the atomic layers is shown on the lower right. The black lines mark the unit cell.

Figure 1 shows the three possible antiferromagnetic ground state configurations within the crystal structure of CaMn$_2$Bi$_2$. The magnetic moments (green and gold arrows) are stabilized on the manganese sites within the plane oriented along the *b*-axis in accord with experimental observations^22^. Our first principles total energy calculations find the Néel-type order to be the ground state consistent with neutron diffraction^22^, with the other candidate magnetic states lying at least 35 meV/Mn above in energy. The magnitude of the magnetic moments along with the band gap and relative total energy of the various magnetic configurations are given in Table 1. Experimentally, the Néel phase exhibits a magnetic moment of 3.85 $\mu _{B}$ and a band gap between 31 and 62 meV, depending on the report^22,28^. Additional recent transport studies find a small activation gap between 2 and 4 meV^24^. Our PBE-based calculations yield a magnetic moment close to the experimental value, but with a zero energy gap. We have also tested the new state-of-the-art density meta-GGA functional SCAN and find a slightly enhanced magnetic moments with a concomitant $\sim 200$ meV band gap. Curiously, this enhancement in band gap and magnetic moments is not found in studies of the cuprates^29–33^, iridates^34^, and 3d perovskite oxides in general^35^.

**Table 1:** Comparison of various theoretically predicted properties for the three possible antiferromagnetic ground states in CaMn$_2$Bi$_2$ using DFT and DFT+*U* ($U=4.75$ eV).

Order	Magnetic ($\mu _{B}$)	Orbital ($\mu _{B}$)	Total ($\mu _{B}$)	Gap (meV)	Relative energy (meV/Mn)
DFT					
Néel	3.923	0.103	4.026	0	0
Stripy	3.926	0.081	4.007	131	35
Zig-Zag	3.981	0.084	4.065	0	108
DFT+U					
Néel	4.606	0.028	4.634	49	0
Stripy	4.591	0.026	4.617	126	22
Zig-Zag	4.596	0.027	4.623	204	42

**Figure 2:**
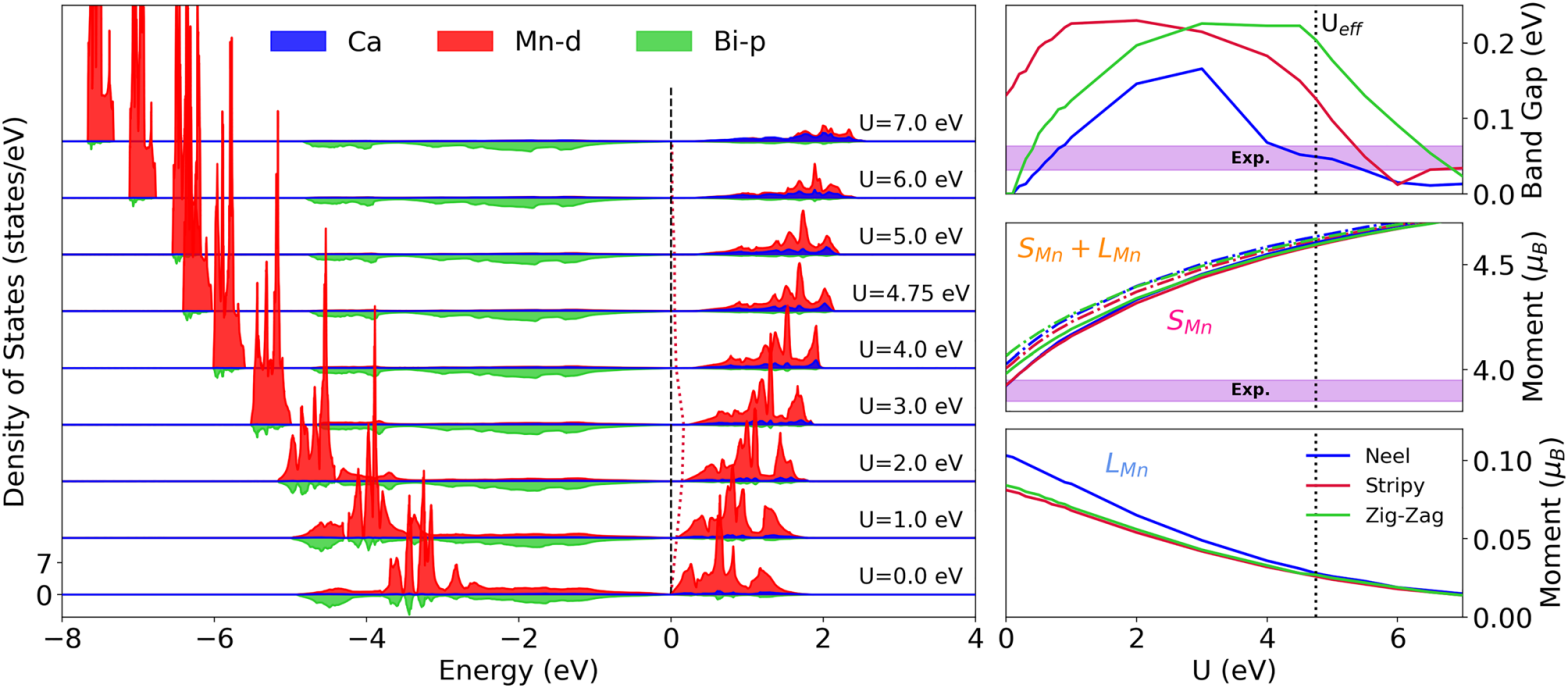
(left panel) Site-resolved partial density of states for the Néel AFM phase for various values of *U*. Shading and lines of various colors (see legend) give the contributions from manganese-*d* and bismuth-*p* orbitals, and Ca atomic weight. The red dashed line marks the leading conduction band edge, with the black dashed line marking the Fermi Energy. For a zoom-in of the band gap see Fig. S4 in the Supplemental Information. (right panel) The spin and orbital (solid lines) components of the total (dot-dashed lines) magnetic moment, along with the band gap as a function of the on-site potential *U*.

To remedy the underestimation of the band gap, we introduce an effective *U* on the Mn-*d* states within the GGA+*U* scheme of Dudarev et al.^36^ To find the *U* that yields the experimental values, a range of on-site Hubbard interaction values were considered. Figure 2 (right panel) shows the evolution of the band gap, the total (dash-dot lines), spin, and orbital (solid lines) magnetic moments as a function of *U* for the various magnetic configurations, along with the average experimental values overlaid (violet shading). Increasing *U* from 0.0 to 2.0 eV a band gap opens in the Néel and Zig-Zag phases, while the gap in the Stripy arrangement increases monotonically. For *U* greater than 3.0 eV, the gap in the Stripy and Néel orders decrease, while that of the Zig-Zag phase flattens. This non-monotonic behavior is observed in a number of oxide materials including MnO$_2$^37^, TiO$_2$^38^ and possibly in the superconducting infinite-layer nickelates^39^, though self-doping effects may be at play in this compound. Simultaneously, the strength of the spin (orbital) magnetic moments continually increase (decrease) with increasing *U*. Since the finite orbital moment is induced on the manganese atom via hybridization with the *p*-orbitals on the bismuth atoms with strong spin-orbit coupling, the decrease in this moment with *U* shows clearly a correlation-induced reduction of hybridization between the atomic species, in favor of electron localization on the Mn site. This localization process concomitantly drives the increase in the size of the spin component of the moment. A *U* of 4.75 eV is found to reproduce the experimentally measured gap value while only enhancing the magnetic moment by $\sim 0.5~\mu _B$, as indicated by the black dotted line. Furthermore, our calculations find the *b*-axis magnetic orientation, as determined by the experiment, to be most stable by 0.1 meV compared to the axis perpendicular to *b*. To cross check our $U_\mathrm{{eff}}$, we calculated the Hubbard *U* derived from the screened $F^0$ Slater integral following the procedure given by Madsen and Novák^40^ as implemented in Wien2k^41^. Within this approach, we find an effective *U* of 4.38 eV on Mn 3*d* electrons, in agreement with our VASP obtained value. Finally, we note these GGA+*U* results improve upon those obtained using the HSE06 hybrid functional^24^.

Figure 2 (left panel) shows the site-resolved density of states (DOS) for various atomic orbitals including Mn-*d*, Bi-*p*, and total Ca weight as a function of the effective *U*. On tuning *U* from 0 to 1 eV the gap in the magnetically split Mn-*d* states (centered at $-3$ eV and 1.0 eV) clearly expands, marking a rise in the Mn-*d* on-site correlations. Importantly, a finite gap opens at the Fermi level, with Mn-*d* dominating the valence and conduction bands. As *U* is increased from 1 to 3 eV, manganese character decreases from the band edges due to the increased on-site correlations. At a Hubbard *U* value of 3 eV, the electronic band gap transitions from Mott-like to a hybridization gap since the low energy state band edge surrounding the Fermi level are composed exclusively of bismuth *p*-orbital character. For *U* > 3 eV the manganese levels continue to move to higher (lower) energies in conduction (valence) band exposing more bismuth density of states. See the Supplemental Information for a detailed close up of the Fermi level. For *U* equal to 4.75 eV, the Mn-*d* states sit slightly above ($\sim 4$ meV) the Bi-*p* levels in the conduction band. We note spin-orbit coupling plays a significant role in reducing the overall electronic band gap, see the Supplemental Information for details. The evolution of the electronic states with on-site correlations clearly demonstrates the critical role the Hubbard *U* parameter plays in achieving the correct delicate balance between correlation and hybridization in this compound.

**Figure 3:**
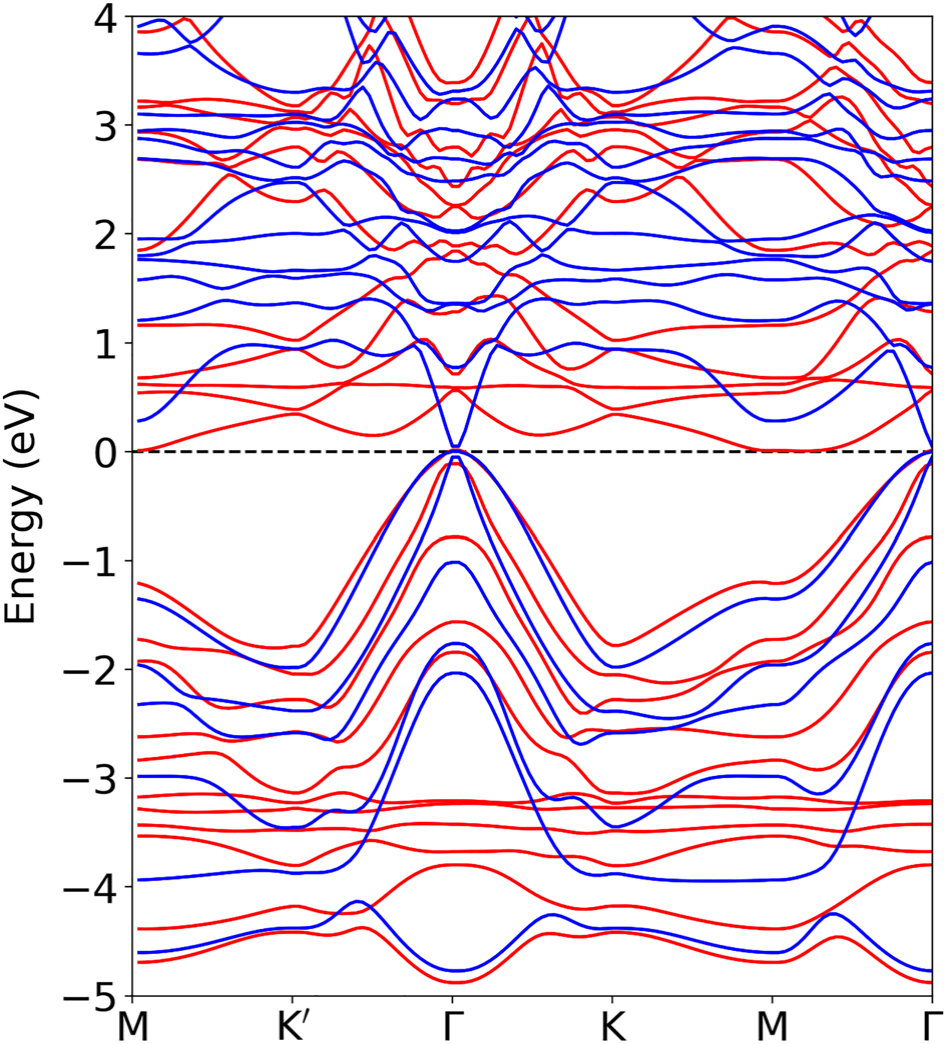
Electronic band dispersion of CaMn$_2$Bi$_2$ in the Néel AFM phase with (blue lines) and without (red lines) an effective on-site potential *U* of 4.75 eV.

Figure 3 shows the electronic band structure of CaMn$_2$Bi$_2$ in the Néel AFM phase with (blue) and without (red) the effective Hubbard ${U}$ of 4.75 eV. Interestingly, the effective on-site correlations appear to change the relative energy of the conical band at $\Gamma$ and the narrow band at *M* in the conduction bands. Specifically, for *U* equal to 4.75 eV, an indirect to direct transition is precipitated, the band extrema of the conduction and valence bands change from $\Gamma$ and *M*, respectively, to centered at $\Gamma$. In the valence states, the band structure resembles that obtained purely by the generalized gradient approximation^22,24^. In contrast, the conduction bands obtained by the HSE06 hybrid functional (Ref.^24^) are more dispersive and display characteristically different transitions, which should be noticeable in optical spectroscopy.

We further note that upon introducing a *U* of 4.75 eV the relative energy between the different AFM ground state configurations has changed. Now we find the Néel and Stripy types to be separated by 22 meV/Mn, while the energy difference to the Zig-Zag ordered state is 42 meV/Mn, making the Stripy and Zig-Zag phases irrelevant in the ground state. For more details see the Supplemental Information.

### Effect of pressure

Applied external pressure provides a direct means to assess the relative ratio between hybridization and correlation strengths in a material. If the band gap is driven by correlations, pressure squeezes the lattice sites of the crystal closer together forcing the wave functions of neighboring atomic sites to overlap. Electrons then tend to become more delocalized in the material, yielding a metal. In contrast, if the band gap is governed by hybridization, pressure further separates bonding and anti-bonding states, thus increasing the band gap. When both hybridization and correlations are present, the gap can undergo non-monotonic behavior under pressure as a result of their competition.

**Figure 4:**
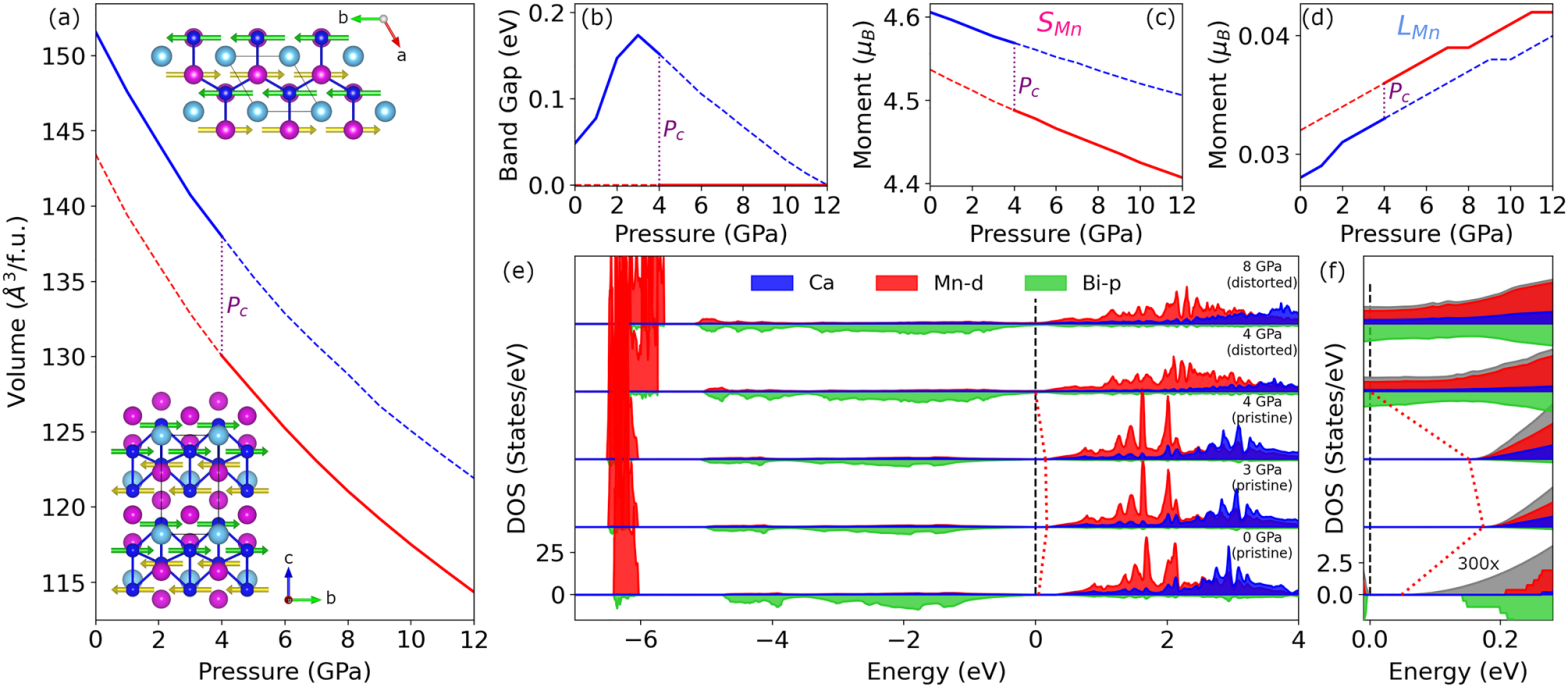
(**a**) Volume per formula unit of CaMn$_2$Bi$_2$ in the Néel type antiferromagnetic phase for both the pristine (blue) and distorted (red) crystal structures using DFT+*U* (*U*=4.75 eV) for various values of pressure. The color of the various atomic species in the crystal structures are the same as in Fig. 1. (**b**) The band gap, along with the (**c**) spin and (**d**) orbital components of the total magnetic moment in the pristine (blue) and distorted (red) crystal structures as a function of pressure. The solid (dashed) lines indicate that the given crystalline phase is stable (unstable). (**e**) Site-resolved partial density of states in the Néel type antiferromagnetic phase of CaMn$_2$Bi$_2$ under various values of pressure. Shading and lines of various colors (see legend) give the contributions from manganese-*d* and bismuth-*p* orbitals, and total Ca atomic weight. Red solid line tracks the leading edge of the conduction states as the system passes through an insulator-metal transition. Black dashed line marks the Fermi Energy. (**f**) Zoom-in of the band gap in the partial density of states in (**e**) with the zero pressure density of states enhanced by 300x to make the tail of the band edge visible. The grey shading and lines indicate the total density of states.

Figure 4e,f shows the site-resolved partial density of states in the Néel-type antiferromagnetic phase of CaMn$_2$Bi$_2$ under hydrostatic pressures from 0 to 8 GPa. At zero pressure the AFM order opens a gap of 49 meV, consistent with the range of reported experimental values. As pressure is applied, the energy separation between the bismuth states surrounding the band gap increases, due to the enhanced hybridization. In contrast, the separation between manganese states above and below the Fermi level decreases. There is also a concomitant slight broadening of the states possibly marking an enhancement of Mn-*d* and Bi-*p* hybridization. In addition, we have calculated the orbital-dependent hybridization function by treating Mn-3*d* as correlated orbitals within the dynamical mean-field theory and find a two-fold increase in the hybridization function, which further corroborates this enhancement in hybridization in the low pressure regime. Furthermore, we have also performed a non-spin-polarized DFT+U calculations to examine the effect of pressure in lieu of spin splitting and also find a marked increase in the direct band gap, indicative of an enhancement of the hybridization with pressure, see the Supplemental Information for details. As the hydrostatic pressure is increased to 3 GPa the Mn-*d* and Bi-*p* levels cross. This changes the character of the conduction band edge from exclusively bismuth to manganese, thereby transforming the compound from a hybridization gap material to a charge-transfer insulator. The pressure of 3 GPa also marks a reversal from positive to negative of the band gap derivative with pressure. The red dashed line follows the evolution of the leading edge of the conduction band and clearly traces out a non-monotonic path with pressure (Fig. 4).

The low pressure behavior of the gap can be rationalized as follows. For small hydrostatic pressures the band gap increases due to the increases in wave function overlap between neighboring atomic lattice sites. However, once pressure becomes too large, the magnetic correlations begin to collapse eventually pulling Mn-3*d* states towards the Fermi level, all while the bismuth levels shift further away from the Fermi energy. Therefore, the hybridization plays a dual role by separating bismuth states at the bands edges, while simultaneously killing magnetic correlations; annihilating the gap. This suggests that CaMn$_2$Bi$_2$ is on the boundary between a correlated charge-transfer insulator and a hybridization gap semiconductor, thereby providing a possible link between the cuprates, pnictides, and heavy fermion systems.

If the pressure is further increased beyond 4 GPa, the planar honeycomb lattice of manganese atoms becomes unstable. To lower the global total energy of the system, a Mn atom slides along the *a*-axis forming a quasi-one-dimensional chain structure. By tracking the enthalpy verses pressure of the two crystalline phases (see the Supplemental Information), we find the structural transition to occur at a critical pressure $P_c$ of 4 GPa in accord with experiment^21^. At this pressure, we predict a dramatic effect on many of the key properties of CaMn$_2$Bi$_2$.

Figure 4a–d shows various key properties of the pristine (blue) and distorted (red) crystal structures for the various pressures, where the relative stability of the two structures is indicated by the solid (stable) and dashed (unstable) lines. At $P_c$, our calculations yield a 8 Å$^3/$f.u. volume collapse across the boundary, while simultaneously, there is an appreciable jump down (up) in the spin (orbital) magnetic moment. Beyond $P_c$ the spin (orbital) magnetic moment steady decreases (increases), again illustrating the competition between hybridization and correlations. The spin magnetization provides a direct indicator of the strength of correlations, while the orbital component tracks the overlap of Bi and Mn atomic wave functions. Furthermore, hybridization between bismuth and manganese atomic wave functions induces an effective spin-orbit coupling on the Mn sites. Finally, this pressure induced plane-to-chain transition also gives way to an insulator to metal transition at $P_c$. We note that theoretical calculations using isotropic expansion and compression were unable to find any energy-equivalent point between the two crystal phases^21^. Here, the mixture of GGA+U and relaxing both the atomic positions and unit cell shape are able to capture the essential physics and lattice degrees of freedom to yield observable results.

Such pressure induced volume collapses can be classified into three categories of mechanisms: correlation driven (e.g. heavy fermion compounds^42^), Jahn-Teller effect (e.g. KCuF$_3$^43^), and Peierls-distortion (e.g. NbSe$_3$^44^). Here, since there is no Fermi surface in the pristine phase no nesting between the various Fermi sheets can occur, ruling out a Peierls-distortion as a possible mechanism. Typically, Jahn-Teller driven effects stem from a change in the *d* electron count. For example, the coordination of the transition metals in the transition metal dichalcogenides varies systematically with *d* electron count between trigonal-prismatic, octahedral, and distorted octahedra (forming quasi-one-dimensional chains)^45^, with the latter distorted structure being characterized as Jahn-Teller driven^46^. For the present material under examination, we only find a marginal increase in the total charge of the Wigner-Seitz sphere, making the Jahn-Teller process unlikely. Therefore, our results suggest that the pressure-induced volume collapse (plane-to-chain) insulator to metal transition is mainly driven by correlation effects.

### Topological character

Originally, CaMn$_2$Bi$_2$ was thought to be a possible magnetic 3D Dirac semimetal, where the Mn-*d* states were assumed to behave as core electrons^47^. This then allows for a clean band inversion of Bi-*s* and Bi-*p* levels. However, Gibson et al.^22^ found that the Mn-*d* orbitals play a significant role at the Fermi level, hybridized with the manifold of bismuth states. This ultimately disrupts the Bi-*s* and Bi-*p* level, avoiding a topological non-trivial ground state.

To confirm the topological nature of CaMn$_2$Bi$_2$ we used the vasp2trace code^48^ in conjunction with the Check Topological Material module^49–51^ provided on the Bilbao Crystallographic Server^52–54^. CaMn$_2$Bi$_2$ is indeed found to be topological trivial for all pressures up to 12 GPa, due to the fact that the Mn-*d* levels dominate the low energy electronic structure and prevent the Bi-*s* and *p* states to overlap and invert. However, if the on-site energy of the manganese bands were to be tuned away from the Fermi level or the Bi-*s* state brought closer to the Fermi level, the bismuth *s* and *p* level could be inverted, making the ground state topologically non-trivial.

## Discussion

In the original analysis performed by Gibson et al.^22^ it was claimed that CaMn$_2$Bi$_2$ behaves as a hybridization gap material. This was justified by tracking only the changes in position of the Bi-*p* and *s* levels with expanding and contracting the unit cell volume by 1% without relaxation, and ignoring the Mn states which cross the Fermi level. Furthermore, after cross checking our results within VASP and Wien2k^41^ we find the electronic structure presented by Gibson et al. to be inconsistent. We believe the bands presented in Ref.^22^ are a result of an incomplete calculation workflow, where the spin-orbit coupling subroutines were possibly neglected. See the Supplemental Information for more details. Additionally, the pressure study by some of us^24^ reports an increase in activation energy of only 20–40 K (2–4 meV) with pressure using electrical transport measurements. The gap observed in Ref.^24^ is one order of magnitude smaller than our prediction, implying the possible existence of impurity states within the gap. The presence of impurities would make the sample sensitive to changes in external pressure and could produce anomalous transport results. Therefore, to accurately compare our first-principles results with the experimental measurements a rigorous modeling of the transport process is required. Additionally, Ref.^24^ reported an increase in $T_N$ with pressure. However, since our density functional theory results only produce the magnetic moment at zero temperature without any measure of the fluctuations, we cannot give any direct insight into the change in $T_N$ with pressure.

## Conclusion

From our analysis of the ground state electronic structure of CaMn$_2$Bi$_2$ as a function of pressure, we find that the low energy electronic structure manifests both correlated charge-transfer insulator and hybridization gap semiconductor characteristics. To fully elucidate its connection to the cuprates, pnictides, and heavy fermion compounds and to what extent they are similar, i.e. exhibiting charge/spin density waves and unconventional superconductivity, further doping dependent studies are needed to uncover its full phase diagram. Moreover, the addition of holes should produce an interesting interplay between itinerant antiferromagnetic carriers and those on the strongly spin-orbit coupled Bi-*p* orbitals, creating a favorable environment for new exotic phases of matter.

## Methods

*Ab initio* calculations were carried out by using the pseudopotential projector-augmented wave method^55^ implemented in the Vienna ab initio simulation package (VASP)^56,57^ with an energy cutoff of 600 eV for the plane-wave basis set. Exchange-correlation effects were treated using the Perdew–Burke–Ernzerhof (PBE) GGA density functional^58^, where a 12 $\times$ 12 $\times$ 8 $\Gamma$-centered k-point mesh was used to sample the Brillouin zone. Spin-orbit coupling effects were included self-consistently at all stages of the calculations. We used the low-temperature zero (high) pressure $P\bar{3}m1$ ($P12_{1}/m1$) crystal structure in accord with the experimental measurements to initialize our calculations^21,59^. The pressure dependence of the various physical properties of CaMn$_2$Bi$_2$ was obtained by increasing (decreasing) the hydrostatic pressure on the zero (high) pressure crystal structure in small quasi-adiabatic steps. For each *U* and pressure, all atomic sites in the unit cell along with the unit cell dimensions were relaxed simultaneously using a conjugate gradient algorithm to minimize energy with an atomic force tolerance of 0.01 eV/Å and a total energy tolerance of $10^{-6}$ eV. The theoretically obtained structural parameters for CaMn$_2$Bi$_2$ in the Néel state at zero pressure, $a=b=4.76$ Å, and $c=7.72$ Å, are in good agreement with the corresponding experimental results.

## Supplementary Information

Supplementary Information.

## Data Availability

All data supporting the findings of this study are available from the corresponding authors upon request.

## References

[1] Nagaosa N (1999). Quantum Field Theory in Strongly Correlated Electronic Systems.

[2] Sun L (2012). Re-emerging superconductivity at 48 Kelvin in iron chalcogenides. Nature.

[3] Stewart G (2011). Superconductivity in iron compounds. Rev. Mod. Phys..

[4] Proust C, Taillefer L (2019). The remarkable underlying ground states of cuprate superconductors. Annu. Rev. Condens. Matter Phys..

[5] Bansil A, Lin H, Das T (2016). Colloquium: Topological band theory. Rev. Mod. Phys..

[6] Takagi H, Takayama T, Jackeli G, Khaliullin G, Nagler SE (2019). Concept and realization of kitaev quantum spin liquids. Nat. Rev. Phys..

[7] Shen B (2020). Strange-metal behaviour in a pure ferromagnetic kondo lattice. Nature.

[8] Kamihara Y, Watanabe T, Hirano M, Hosono H (2008). Iron-based layered superconductor La [O_1-x_ F_x_] FeAs (x = 0.05–0.12) with Tc= 26 k. JACS.

[9] Wen H-H, Li S (2011). Materials and novel superconductivity in iron pnictide superconductors. Annu. Rev. Condens. Matter Phys..

[10] Wang C (2008). Thorium-doping-induced superconductivity up to 56 k in Gd_1- x_Th_x_FeAso. EPL.

[11] Yanagi H (2009). Antiferromagnetic bipolar semiconductor LaMnPO with ZrCuSiAs-type structure. J. Appl. Phys..

[12] McGuire MA, Garlea VO (2016). Short-and long-range magnetic order in LaMnAso. Phys. Rev. B.

[13] Zhang Q (2016). Structure and magnetic properties of Ln MnSbO (Ln = La and Ce). Phys. Rev. B.

[14] Simonson J (2012). From antiferromagnetic insulator to correlated metal in pressurized and doped LaMnPO. Proc. Natl. Acad. Sci..

[15] Simonson J (2011). Gap states in insulating LaMnPO_1–x_ F_x_ (x = 0–0.3). Phys. Rev. B.

[16] Sun Y-L (2012). Insulator-to-metal transition and large thermoelectric effect in La_1–x_Sr_x_MnAsO. EPL.

[17] Hanna T (2013). From antiferromagnetic insulator to ferromagnetic metal: Effects of hydrogen substitution in LaMnAsO. Phys. Rev. B.

[18] Cheng J-G (2015). Pressure induced superconductivity on the border of magnetic order in MnP. Phys. Rev. Lett..

[19] Chong X, Jiang Y, Zhou R, Feng J (2016). Pressure dependence of electronic structure and superconductivity of the Mn_x_ (x = N, P, As, Sb). Sci. Rep..

[20] Kawaguchi N, Urata T, Hatano T, Iida K, Ikuta H (2018). Nonmonotonic and anisotropic magnetoresistance effect in antiferromagnet caMn_2_Bi_2_. Phys. Rev. B.

[21] Gui X (2019). Pressure-induced large volume collapse, plane-to-chain, insulator to metal transition in CaMn_2_Bi_2_. Inorg. Chem..

[22] Gibson Q (2015). Magnetic and electronic properties of CaMn_2_Bi_2_: A possible hybridization gap semiconductor. Phys. Rev. B.

[23] Zada Z, Laref A, Murtaza G, Zeb A, Yar A (2019). First-principles calculations of electronic and magnetic properties of *X*Mn_2_*Y*_2_ (*X* = Ca, Sr; *Y* = Sb, Bi) compounds. Int. J. Modern Phys. B.

[24] Piva M (2019). Putative hybridization gap in CaMn_2_ Bi_2_ under applied pressure. Phys. Rev. B.

[25] Hundley M, Canfield P, Thompson J, Fisk Z, Lawrence J (1990). Hybridization gap in Ce_3_Bi_4_Pt_3_. Phys. Rev. B.

[26] Cooley J, Aronson M, Canfield P (1997). High pressures and the kondo gap in Ce_3_Bi_4_Pt_3_S. Phys. Rev. B.

[27] Campbell, D. J. *et al.* High pressure kondo insulator-semimetal transition in Ce_3_Bi_4_Pt_3_. arXiv:1907.09017 (2019).

[28] Sangeetha N, Smetana V, Mudring A-V, Johnston D (2018). Antiferromagnetism in semiconducting SrMn_2_Sb_2_ and BaMn_2_Sb_2_ single crystals. Phys. Rev. B.

[29] Furness JW (2018). An accurate first-principles treatment of doping-dependent electronic structure of high-temperature cuprate superconductors Nat. Commun. Phys..

[30] Lane C (2018). Antiferromagnetic ground state of La_2_CuO_4_: A parameter-free ab initio description. A. Phys. Rev. B.

[31] Zhang Y (2020). Competing stripe and magnetic phases in the cuprates from first principles. Proc. Natl. Acad. Sci..

[32] Nokelainen J (2020). *Ab initio* description of the Bi_2_Sr_2_CaCu_2_O_8+δ_ electronic structure. Phys. Rev. B.

[33] Pokharel, K. *et al.**Ab intio* description of the electronic structure of high-temperature cuprate superconductors: A comparative density functional study. *arXiv preprint* arXiv:2004.08047 (2020).

[34] Lane C (2020). First-principles calculation of spin and orbital contributions to magnetically ordered moments in Sr_2_IrO_4_. Phys. Rev. B.

[35] Varignon J, Bibes M, Zunger A (2019). Origin of band gaps in 3*d* perovskite oxides. Nat. Commun..

[36] Dudarev S, Botton G, Savrasov S, Humphreys C, Sutton A (1998). Electron-energy-loss spectra and the structural stability of nickel oxide: An LSDA+U study. Phys. Rev. B.

[37] Wang Y-C, Chen Z-H, Jiang H (2016). The local projection in the density functional theory plus U approach: A critical assessment. J. Chem. Phys..

[38] Han X (2013). Limitation and extrapolation correction of the GGA+U formalism: A case study of Nb-doped anatase TiO_2_. J. Mater. Chem. C.

[39] Lee K-W, Pickett W (2004). Infinite-layer LaNiO_2_: Ni^1+^ is not Cu^2+^. Phys. Rev. B.

[40] Madsen GKH, Novák P (2005). Charge order in magnetite: An LDA+U study. EPL Europhys. Lett..

[41] Blaha, P. *et al.* wien2k An augmented plane wave+local orbitals program for calculating crystal properties (2001).

[42] Lipp MJ (2017). Anomalous elastic properties across the $$\gamma$$ to $$\alpha$$ volume collapse in cerium. Nat. Commun..

[43] Kugel KI, Khomskii D (1982). The jahn-teller effect and magnetism: Transition metal compounds Soviet. Phys. Uspekhi.

[44] Boswell F, Prodan A (1980). Peierls distortions in NbS_3_ and NbSe_3_. Phys. B+C.

[45] Wilson JA, Yoffe A (1969). The transition metal dichalcogenides discussion and interpretation of the observed optical, electrical and structural properties. Adv. Phys..

[46] Kertesz M, Hoffmann R (1984). Octahedral vs. trigonal-prismatic coordination and clustering in transition-metal dichalcogenides. J. Am. Chem. Soc..

[47] Zhang T, Jiang Y, Song Z, Huang H, He Y, Fang Z, Weng H, Fang C (2019). Catalogue of topological electronic materials. Nature.

[48] Vergniory M, Elcoro L, Felser C, Regnault N, Bernevig BA, Wang Z (2019). A complete catalogue of high-quality topological materials. Nature.

[49] Bradlyn B, Elcoro L, Cano J, Vergniory M, Wang Z, Felser C, Aroyo M, Bernevig BA (2017). Topological quantum chemistry. Nature.

[50] Vergniory M, Elcoro L, Wang Z, Cano J, Felser C, Aroyo M, Bernevig BA, Bradlyn B (2017). Graph theory data for topological quantum chemistry. Phys. Rev. E.

[51] Elcoro L (2017). Double crystallographic groups and their representations on the bilbao crystallographic server. J. Appl. Crystallogr..

[52] Aroyo MI (2011). Crystallography online: Bilbao crystallographic server Bulg. Chem. Commun..

[53] Aroyo MI (2006). Bilbao crystallographic server: I: Databases and crystallographic computing programs. Cryst. Mater..

[54] Aroyo MI, Kirov A, Capillas C, Perez-Mato J, Wondratschek H. Bilbao (2006). crystallographic server: ii—Representations of crystallographic point groups and space groups. Acta Crystallogr. A.

[55] Kresse G, Joubert D (1999). From ultrasoft pseudopotentials to the projector augmented-wave method. Phys. Rev. B.

[56] Kresse G, Furthmüller J (1996). Efficient iterative schemes for *ab initio* total-energy calculations using a plane-wave basis set. Phys. Rev. B.

[57] Kresse G, Hafner J (1993). *Ab initio* molecular dynamics for open-shell transition metals. Phys. Rev. B.

[58] Perdew JP, Burke K, Ernzerhof M (1996). Generalized gradient approximation made simple. Phys. Rev. Lett..

[59] Cordier G, Schäfer H, Naturforsch Z (1976). Neue intermetallische verbindungen im anti-Ce_2_O_2_S-strukturtyp/new intermetallic compounds in the anti-Ce_2_O_2_S-structure type. B Chem. Sci..

