# Supplementary material for: Correlation versus hybridization gap in CaMn2Bi2
Source: Sci Rep. 2023 Jun 7;13:9271. doi: 10.1038/s41598-023-35812-2 (PMC10247774; doi:10.1038/s41598-023-35812-2)
Supplement: Supplementary file 1 — Supplementary Information. [file 41598_2023_35812_MOESM1_ESM.pdf]

Supplementary Information:
Correlation versus Hybridization Gap in $CaMn_2Bi_2$

Christopher Lane,[1,2] M. M. Piva,[3,4] P. F. S. Rosa,[5] and Jian-Xin Zhu[1,2]

[1] *Theoretical Division, Los Alamos National Laboratory, Los Alamos, New Mexico 87545, USA*
[2] *Center for Integrated Nanotechnologies, Los Alamos National Laboratory, Los Alamos, New Mexico 87545, USA*
[3] *Max Planck Institute for Chemical Physics of Solids, Nöthnitzer Str. 40, D-01187 Dresden, Germany*
[4] *Institudo de Física "Gleb Wataghin", UNICAMP, 13083-859, Campinas, SP, Brazil*
[5] *Division of Materials Physics and Application, Los Alamos National Laboratory, Los Alamos, New Mexico 87545, USA*

(Dated: May 17, 2023)

RELATIVE ENERGY, BAND GAP, AND MAGNETIC MOMENTS AS A FUNCTION OF U

Figure S1 shows the relative energy (a), band gap (b), and magnitude of the Mn spin (c) and orbital (d) magnetic moments for $CaMn_2Bi_2$ in various magnetic phases as a function of Hubbard U. Data for both the experimental (dashed line) and relaxed (solid line) crystal structure are presented. For all U studied the Néel AFM order is the ground state, with the Stripy and Zig-Zag configurations lying above 20 meV/Mn and 40 meV/Mn for all values of U, respectively. Interestingly the band gap is highly sensitive to the relaxation of the lattice and atomic positions, displaying changes on the order of 100 meV depending on the magnetic order and value of U.

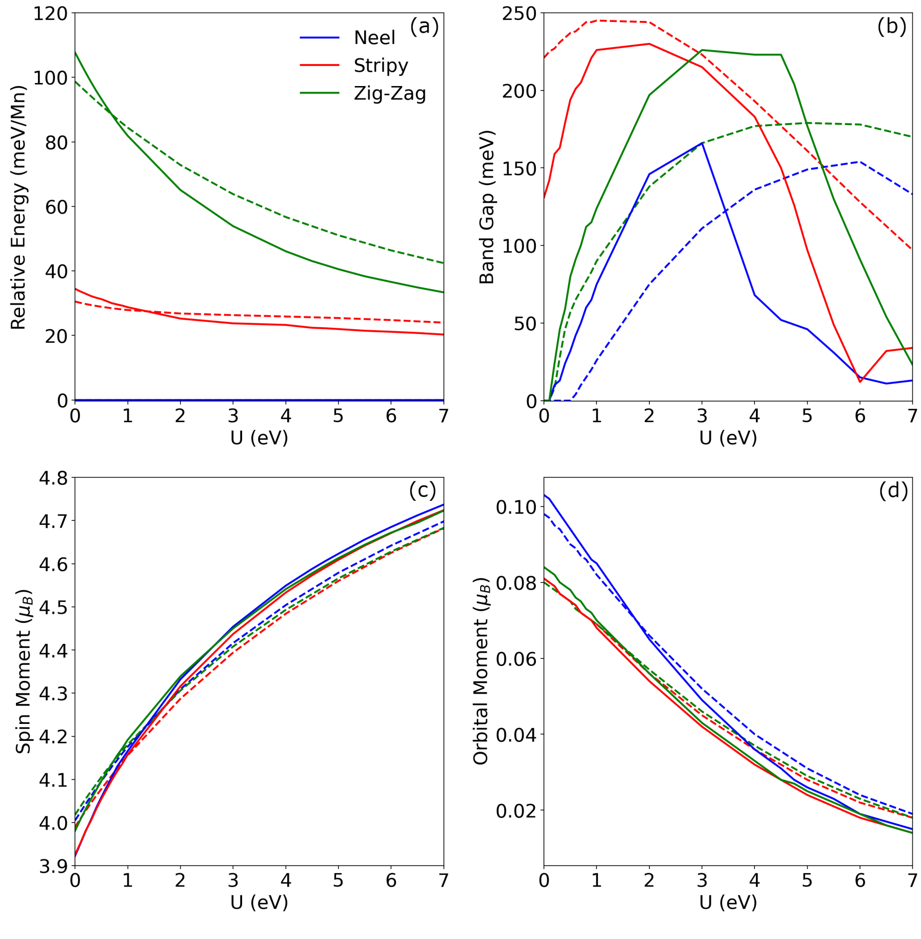

FIG. S1. (color online) Relative energy (a), electronic band gap (b), and Mn magnetic moments (c-d) of $CaMn_2Bi_2$ in the Néel, Stripy, and Zig-Zag magnetic phases as a function of Hubbard U. The solid lines (dashed lines) are for the fully relaxed (experimental) crystal structure.

RELATIVE ENTHALPY, ENERGY-VOLUME CURVES, BAND GAP, AND MAGNETIC MOMENTS AS A FUNCTION OF PRESSURE

Figure S2 shows the relative enthalpy (a), Energy-volume curves (b), band gap (c), and magnitude of the Mn spin (d) and orbital (e) magnetic moments for $CaMn_2Bi_2$ in various magnetic phases as a function of hydrostatic pressure. Data for both the distorted (dashed line) and pristine (solid line) crystal structures are presented. For all pressures studied the Néel AFM order is the ground state irrespective of the Mn distortion. In the pristine case, the Stripy and Zig-Zag configurations lie $\sim$ 20 meV/Mn and $\sim$ 50 meV/Mn above for all pressures, respectively, whereas in the distorted phase the Stripy and Zig-Zag phases are nearly degenerate ($\sim$ 1 meV/Mn) lying $\sim$ 10 meV/Mn above the Néel state. For low pressures ($P < 3$ GPa), the band gap is highly sensitive to the magnetic order. Specifically, that of the Néel AFM order increases with increased pressure until 3 GPa, where the gap begin to decrease. This behavior is clearly indicative of a hybridization gap for $P < 3$ GPA and a correlation gap for $P > 3$ GPa. Interestingly, the Stripy and Zig-Zag phases display a predominantly correlation gap behavior.

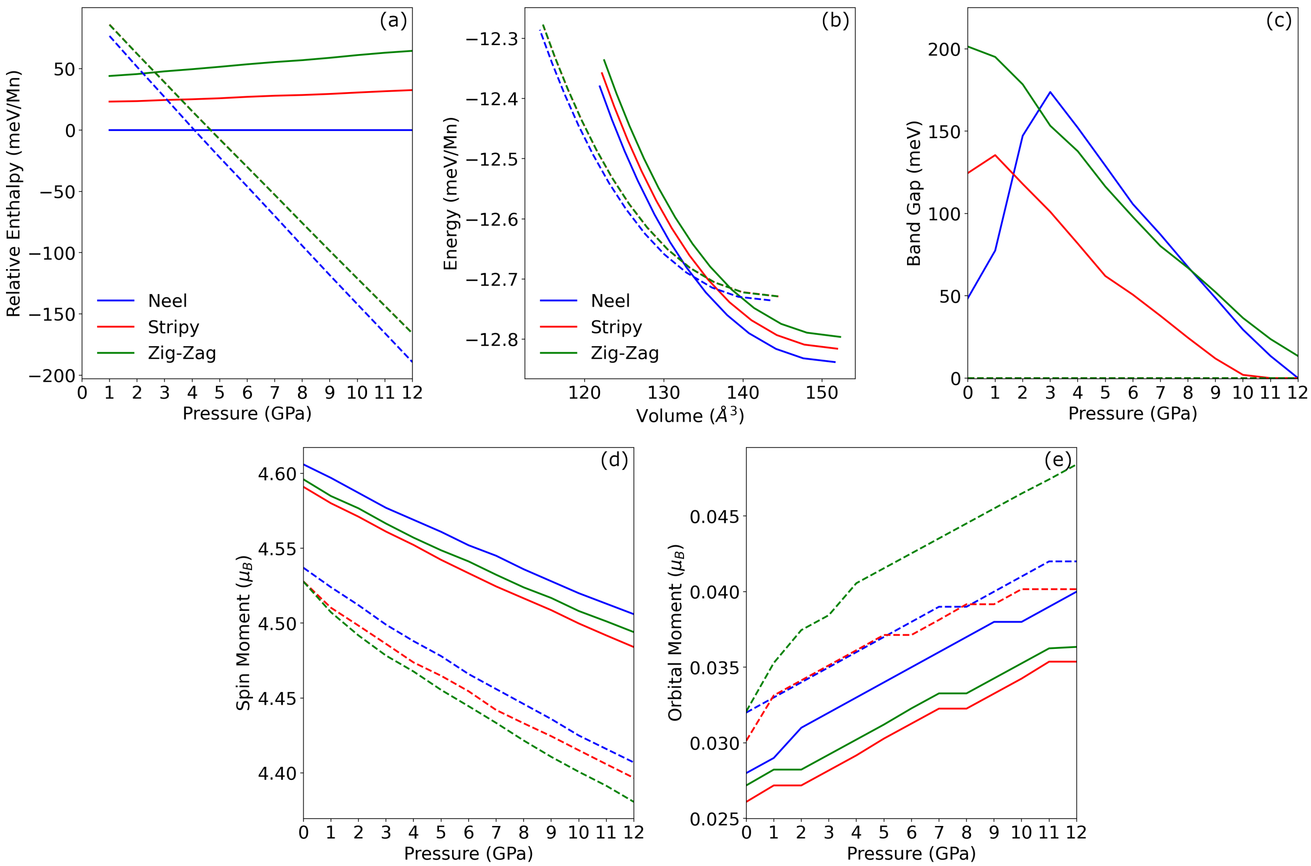

FIG. S2. (color online) Relative enthalpy (a), Energy-volume curves (b), electronic band gap (b), and Mn magnetic moments (c-d) of $CaMn_2Bi_2$ in the Néel, Stripy, and Zig-Zag magnetic phases as a function of hydrostatic pressure. The solid lines (dashed lines) are for the pristine (distorted) crystal structure.

ELECTRONIC BAND STRUCTURE AND DENSITY OF STATES WITH AND WITHOUT SPIN-ORBIT COUPLING

Figure S3 shows the electronic band structure and density of states with (a) and without (b) spin-orbit coupling for $CaMn_2Bi_2$ in the Néel magnetic phase with a Hubbard U of 4.75 eV. The effect of spin-orbit coupling appears to be strongest about the band gap, where the gap is slightly larger when spin-orbit coupling is turned off. Furthermore, there is marginal reorganization of the peak structure of the density of states.

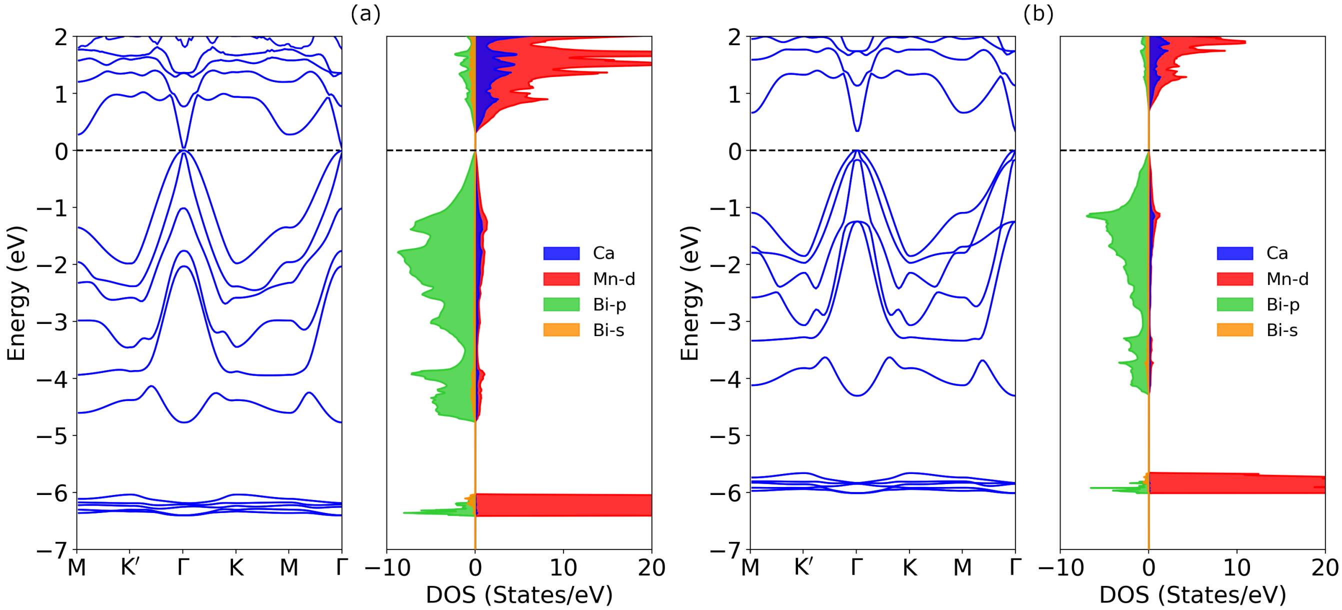

FIG. S3. (color online) Electronic band structure and density of states with (a) and without (b) spin-orbit coupling for $CaMn_2Bi_2$ in the Néel magnetic phase with a Hubbard U of 4.75 eV.

ELECTRONIC DENSITY OF STATES FOR VARIOUS HUBBARD U VALUES

Figure S4 presents the full and zoomed-in site-resolved partial density of states of $CaMn_2Bi_2$ in the Néel AFM phase for various values of U. Shading and lines of various colors (see legend) give the contributions from manganese-d and bismuth-p orbitals, Ca atomic weight, and the total density of states.

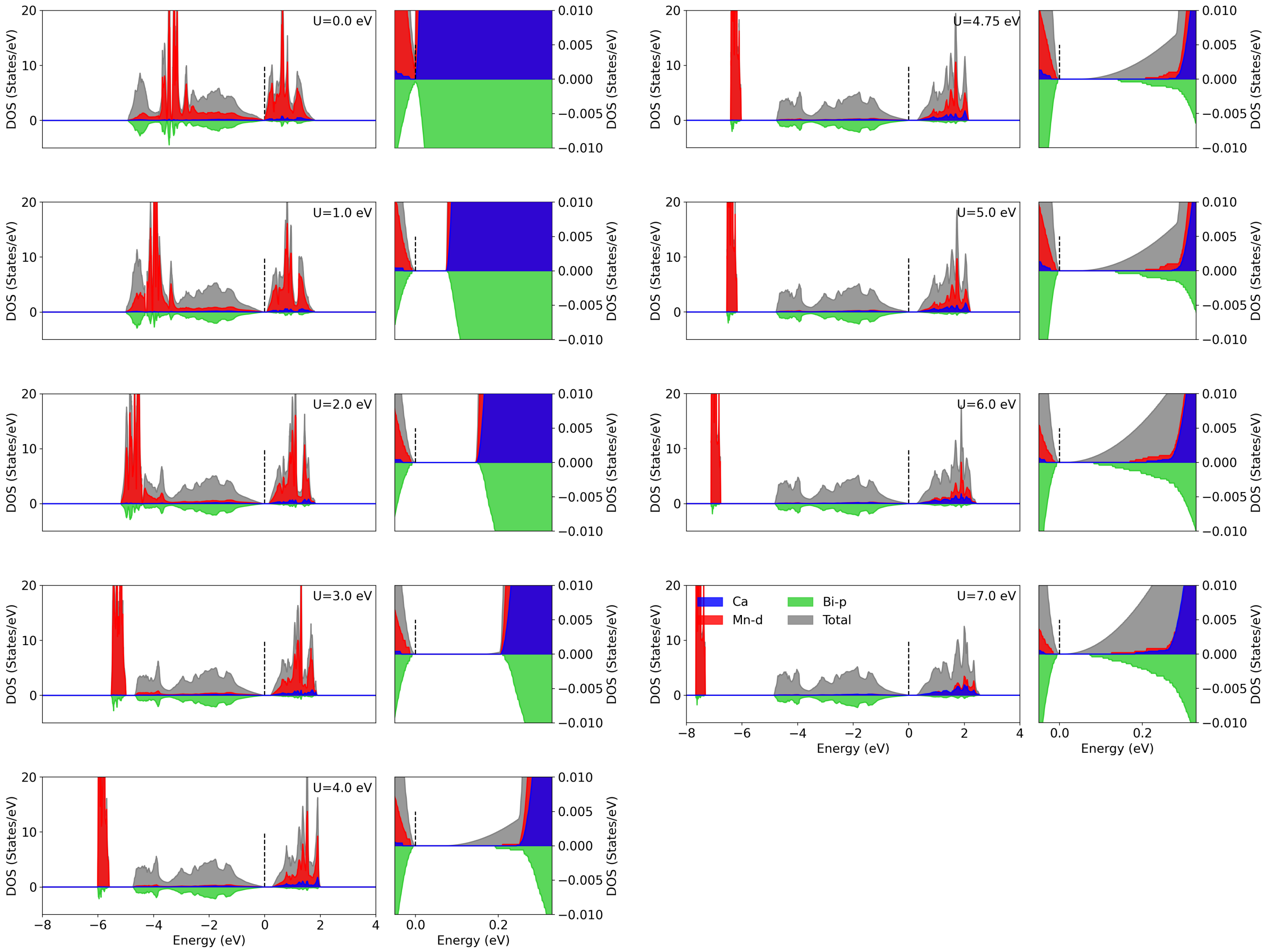

FIG. S4. (color online) Site-resolved partial density of states of $CaMn_2Bi_2$ in the Néel AFM phase for various values of U for the full energy range and a close-up on the band gap.

ELECTRONIC BAND STRUCTURE FOR VARIOUS PRESSURES

Figure S5 shows the grand state electronic bands structure at various pressures. A insulating-metallic transition is clearly observed across the critical pressure separating pristine and distorted equilibrium crystal structures.

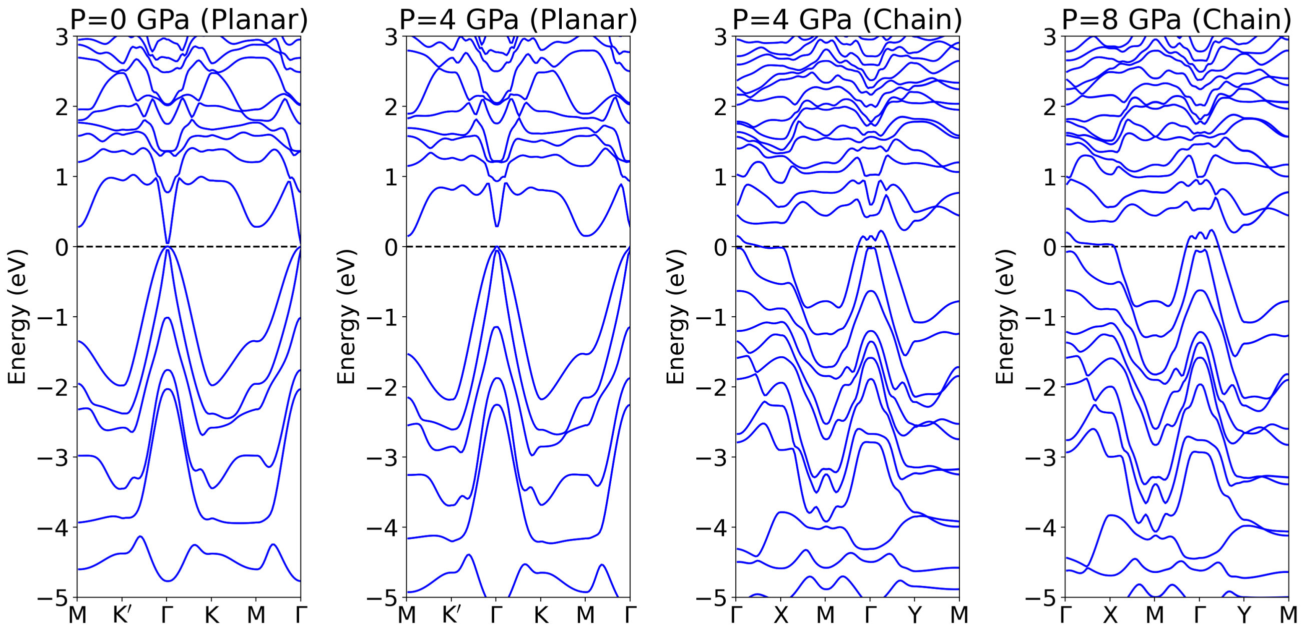

FIG. S5. (color online) Electronic band structure of $CaMn_2Bi_2$ in the ground state Néel phase for pressure below and above the structural transition at $P_c = 4$ GPa.

WIEN2K BAND STRUCTURE BENCHMARK

We have performed a benchmark band structure calculation within Wien2k for a large $U = 7$ eV to compare to Ref. 22. Figure S6 presents the electronic band structure of $CaMn_2Bi_2$ in the ground state Néel phase for a U of 7 eV. We find the resulting bands to be consistent with our results obtained from VASP. The workflow for calculating the band structure in Wien2k has a number of steps which must be taken in proper order, when including spin-orbit coupling and orbital-dependent potential due to the Hubbard U. We suspect the band structure calculation in Ref. 22 has missed a critical step in Wien2k workflow when generating the band dispersions.

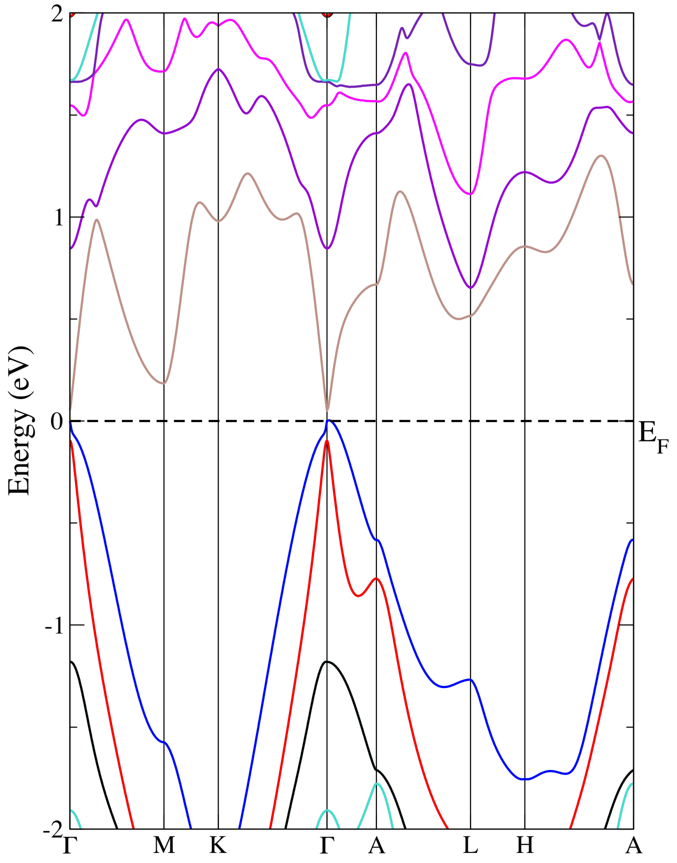

FIG. S6. (color online) Electronic band structure of $CaMn_2Bi_2$ in the ground state Néel phase for a U of 7 eV.

BRILLOUIN ZONE HIGH-SYMMETRY K-POINT DEFINITIONS FOR THE PRISTINE AND DISTORTED CRYSTALS

Figure S7 shows the Brillouin Zone with the high-symmetry k-points labeled for the (a) pristine and (b) distorted crystal structure of $CaMn_2Bi_2$.

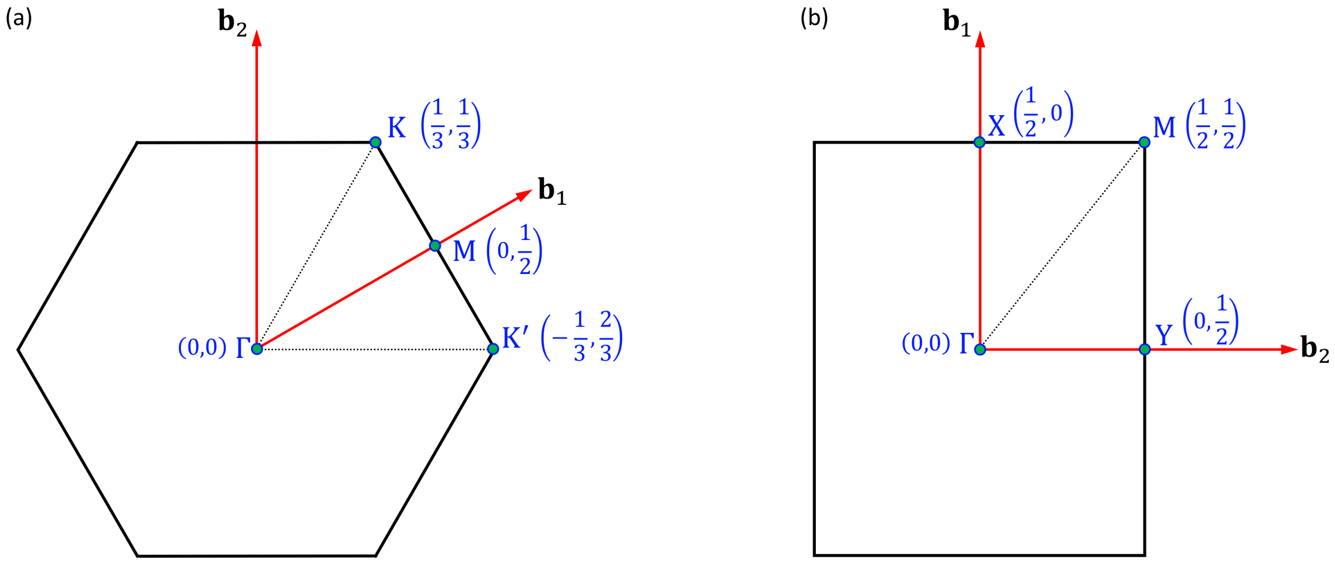

FIG. S7. (color online) Brillouin Zone with the high-symmetry k-points indicated for the (a) pristine and (b) distorted crystal structure of $CaMn_2Bi_2$.

HYBRIDIZATION FUNCTION FOR VARIOUS PRESSURES

Figure S8 shows the hybridization function for both Mn sites (right and left panels) in AFM $CaMn_2Bi_2$ evaluated at the Fermi level

$$\Delta_{\mu\sigma}(w=0) = \sum_{n\mathbf{k}} \frac{|V_{n\mathbf{k}}^{\mu\sigma}|^2}{-\varepsilon_{n\mathbf{k}} + i\delta},$$

where $V_{n\mathbf{k}}^{\mu\sigma}$ describes the hybridization between the magnetic site and the bath, $\varepsilon_{n\mathbf{k}}$ are the non-correlated electronic levels of the bath, and we have analytically continued from Matsubara to real frequencies by $i\omega_n \to \omega + i\delta$. The orbitals d_{xz} and d_{yz} ($d_{x^2-y^2}$ and d_{xy}) are found to be degenerate due to crystal symmetry. The pair of hybridization channels $d_{x^2-y^2}$ and d_{xy} dominate, with the remaining manifold of states lying about a factor of 2 below. As pressure is increased from 0 GPa to 4 GPa the value of the hybridization function doubles in value irrespective of orbital or spin configuration. This clearly demonstrates the key role hybridization plays at the Fermi level for pressures below the structural transition.

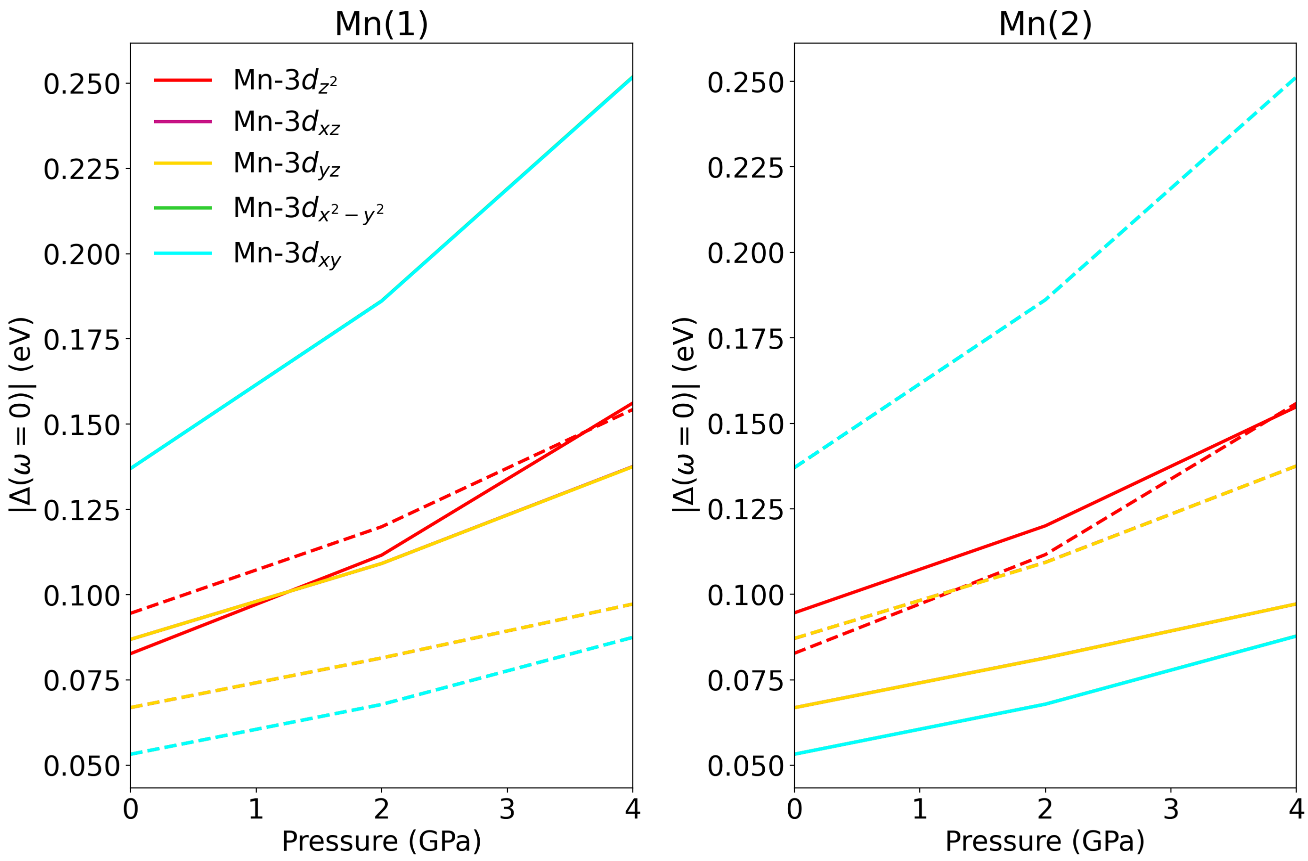

FIG. S8. (color online) Hybridization function of $CaMn_2Bi_2$ in the ground state Néel phase for low pressures and various orbitals as indicated. The solid lines (dashed lines) denote the spin-up (spin-down) component of $\Delta(\omega=0)$. Note, orbitals d_{xz} and d_{yz} ($d_{x^2-y^2}$ and d_{xy}) are degenerate.

NON-SPIN-POLARIZED DFT+U CALCULATIONS

Figure S9 shows the electronic band structure of pristine $CaMn_2Bi_2$ in the ground state Néel phase for a U of 4.75 eV obtained by non-spin-polarized DFT+U calculations for various low pressures. The bands near the Fermi level are found to become more dispersive with increased pressure, which is also accompanied by an increase of direct band gap. This is inline with the observed increase in the hybridization function buttressing the key role hybridization plays at the Fermi level for low pressures leading to the structural transition at 4 GPa. We have also performed non-spin-polarized DFT+U calculations within Wien2k and find the same trend.

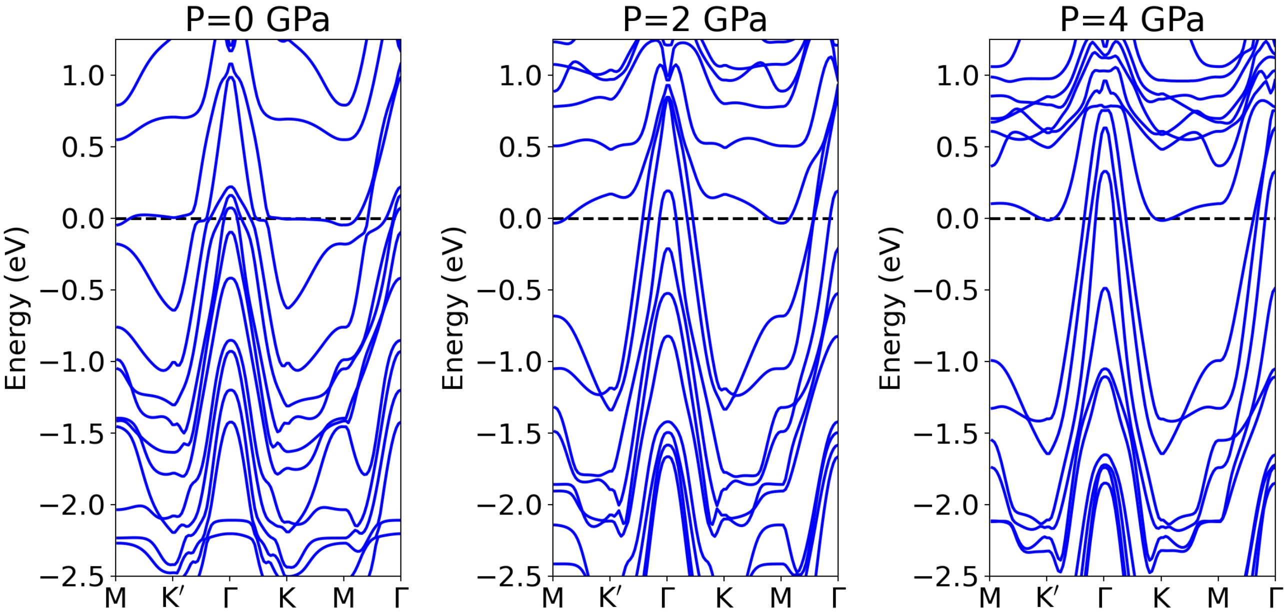

FIG. S9. (color online) the electronic band structure of pristine $CaMn_2Bi_2$ in the ground state Néel phase for a U of 4.75 eV obtained by non-spin-polarized DFT+U calculations for various low pressures indicated.
